# Oleanolic acid induces p53-dependent apoptosis *via* the ERK/JNK/AKT pathway in cancer cell lines in prostatic cancer xenografts in mice

**DOI:** 10.18632/oncotarget.25316

**Published:** 2018-05-29

**Authors:** Gyeong-Ji Kim, Hyeon-Ju Jo, Kwon-Jai Lee, Jeong Woo Choi, Jeung Hee An

**Affiliations:** ^1^ Department of Biomedical Engineering, Sogang University, Seoul, Republic of Korea; ^2^ Department of Food Science and Technology, Seoul National University of Science & Technology, Seoul, Republic of Korea; ^3^ Department of Advanced Materials Engineering, Daejeon University, Daejeon, Republic of Korea; ^4^ Department of Chemical and Biomolecular Engineering, Sogang University, Seoul, Republic of Korea; ^5^ Division of Food Bioscience, Konkuk University, Chunju, Korea

**Keywords:** anticancer activity, apoptosis, oleanolic acid, cell cycle arrest, MAPK signaling

## Abstract

We evaluated oleanolic acid (OA)-induced anti-cancer activity, apoptotic mechanism, cell cycle status, and MAPK kinase signaling in DU145 (prostate cancer), MCF-7 (breast cancer), U87 (human glioblastoma), normal murine liver cell (BNL CL.2) and human foreskin fibroblast cell lines (Hs 68). The IC50 values for OA-induced cytotoxicity were 112.57 in DU145, 132.29 in MCF-7, and 163.60 in U87 cells, respectively. OA did not exhibit toxicity in BNL CL. 2 and Hs 68 cell lines in our experiments. OA, at 100 µg/mL, increased the number of apoptotic cells to 27.0% in DU145, 27.0% in MCF-7, and 15.7% in U87, when compared to control cells. This enhanced apoptosis was due to increases in p53, cytochrome c, Bax, PARP-1 and caspase-3 expression in DU145, MCF-7 and U87 cell lines. OA-treated DU145 cells were arrested in G2 because of the activation of p-AKT, p-JNK, p21 and p27, and the decrease in p-ERK, cyclin B1 and CDK2 expression; OA-treated MCF-7 cells were arrested in G1 owing to the activation of p-JNK, p-ERK, p21, and p27, and the decrease in p-AKT, cyclin D1, CDK4, cyclin E, and CDK2; and OA-treated U87 cells also exhibited G1 phase arrest caused by the increase in p-ERK, p-JNK, p-AKT, p21, and p27, and the decrease in cyclin D1, CDK4, cyclin E and CDK2. Thus, OA arrested the cell cycle at different phases and induced apoptosis in cancer cells. These results suggested that OA possibly altered the expression of the cell cycle regulatory proteins differently in varying types of cancer.

## INTRODUCTION

Cancer represents one of the major health problems in the world today [1]. In 2008, approximately 12.7 million cancers were reported worldwide and this number was estimated to increase to 21 million by 2030 [2]. According to the National Cancer Information Center in Korea, cancer occurrences were more prevalent in males when compared to females [3]. In 2014, cancer incidences in Korea were reported to be the highest for the thyroid gland, followed by other organs such as the stomach, colon, breast, lung, and prostate [3]. Chemotherapy is effective against most cancer types, but drug resistance limits the success of chemotherapy in many cases, and inability of chemotherapeutic drugs to distinguish between normal and cancerous cells hinders their applicability [4, 5]. Generally, resistance to anticancer drugs is a complex and multifactorial phenomenon [4, 5]. Many factors are also involved in drug sensitivity; these factors include drug efflux, drug inactivation, alterations of drug targets, DNA methylation, adaptation, and restraint of damage induced by drugs, cell cycle arrest, and apoptosis [4]. Hundreds of compounds have been found to modulate the drug-resistant phenotypes *in vitro*; however, their clinical applications remained limited owing to high toxicity *in vivo* [5]. Thus, utmost attention is being given to searching for better and safer drugs of natural origin, which may potentially increase the efficacy of anticancer treatments [5].

Apoptosis, or programmed cell death, is the most common mechanism used to induce cancer cell death via targeted chemotherapy [6]. It is a regulated process that is activated by stressors such as DNA damage, cytokines, and oxidative stress [7]. The p53 tumor suppressor is activated by the oncogene- or DNA damage-induced signaling pathways, which in turn accelerates the transcription of several genes involved in apoptosis such as the proapoptotic members of the Bcl-2 family, including those encoding for death receptors [8]. Bax is an important proapoptotic member of the Bcl-2 family of proteins that regulates the balance between cell survival and death [9]. In response to apoptotic signals, Bax is transformed into a fatal mitochondrial oligomer that causes mitochondrial damage, representing an important step for the intrinsic apoptotic pathway [10, 11]. Additionally, p53-induced apoptosis also activates caspases [8], primarily occurring through the activation of the death receptor pathway or through mitochondrial membrane depolarization [6].

The relationship between the cell cycle and apoptosis is underscored by the role of the p53 tumor suppressor gene and those of the p21WAF1/CIP1 and *BAX* genes, which induce cell cycle arrest and cell death [12]. Cell proliferation is mediated by several signaling molecules and checkpoints that regulate cell division [13]. The progression through the cell cycle is positively regulated by cyclin E and the cyclin-dependent kinase (CDK) complex, which phosphorylate the retinoblastoma tumor suppressor protein to induce the transition from the G1 to the S phase [10]. However, the p21WAF1/CIP1 and p27KIP1 kinase inhibitor proteins bind to the cyclin E/CDK2 complex and block the G1/S transition [14]. I Another protein, cyclin B1, also plays a key role in the cell cycle transition from the G2 to M phase [15], and the decrease in its expression levels has been suggested to disrupt cell growth and promote malignant transcription [16].

Oleanolic acid (3-β-hydroxy-olea-12-en-28-oic acid; OA) is a naturally occurring pentacyclic triterpenoic acid [17, 18] that exhibits chemopreventive, hepatoprotective, tumor-suppressive, contraceptive, anti-inflammatory, antioxidant, antimicrobial, antiparasitic, antiviral, and antineoplastic characteristics [19–23]. The tumor-suppressive activity of OA was demonstrated in several cancer cell lines such as KB, HT29, MCF-7, MDA-MA-231, HCT-116, HONE-1, Hep-G2, and HL-60 [20, 24–26]. Recently, several reports showed that OA also induced G1 cell cycle arrest in the GBC-SD, NOZ, HCT15, and K562 cell lines [21, 27]. Moreover, it was reported that OA induced a concentration-dependent S phase and G2/M phase cell cycle arrest in Panc-28 and Hep-G2 cells [28, 29]. The inhibitory effects of OA were attributed to the suppression of specific intracellular signaling pathways such as the STAT3, JNK, AKT, and NF-kappaB [30]. As a result, these studies proposed OA as an adjunct to cancer chemotherapy.

In this study, we investigated the cellular viability, apoptotic process, and cell cycle in OA-treated DU145 (prostate cancer), MCF-7 (breast cancer), and U87 (human glioblastoma) cells. Also, DU145 cell xenografts grown in BALB/C mice were injected with OA. We explored the protein expression of apoptosis, cell cycle and kinase signaling in DU145 cell xenografts grown in mice treated OA. Additionally, we also examined protein expression with respect to apoptosis, cell cycle, and kinase signaling in these OA-treated cells. The results suggested that OA differentially altered the expression of cell cycle regulatory proteins depending on the type of cancer cells.

## RESULTS

### Cytotoxic activity of OA in cancer cells

To investigate the effects of OA on cell viability in cancer cell lines (DU145, MCF-7, U87), the cells were treated with 0, 25, 50, 100, and 250 µg/mL OA for 24 h and cell proliferation was assessed using the MTT assay (Table 1). Normal, non-cancerous cell lines such as BNL CL. 2 (murine liver cells) and Hs 68 (human foreskin fibroblast) were also added to the experiment as a means of positive control. While the IC_50_ values (inhibitory concentration 50), following OA treatment, were reported as 112.57, 132.29, and 163.60 µg/mL in DU145, MCF-7, and U87 cell lines respectively, OA did not appear to exhibit toxicity in BNL CL. 2 and Hs 68 cell lines, even at concentrations greater than 1000 µg/mL. Therefore, we sought to investigate the underlying molecular mechanism of the anti-tumor effect OA exhibited on cancer cells.

**Table 1:** Cytotoxicity exhibited by oleanolic acid (OA) in cancer cell lines (DU145, MCF-7, U87, Hs 68, and BNL CL2) as determined by the MTT assay

Cell lines	DU145	MCF-7	U87	Hs 68	BNL CL.2
IC_50_ (μg/mL)	112.57	132.29	163.60	>1000	>1000

### Proapoptotic activity of OA in cancer cells

**Figure 1:**
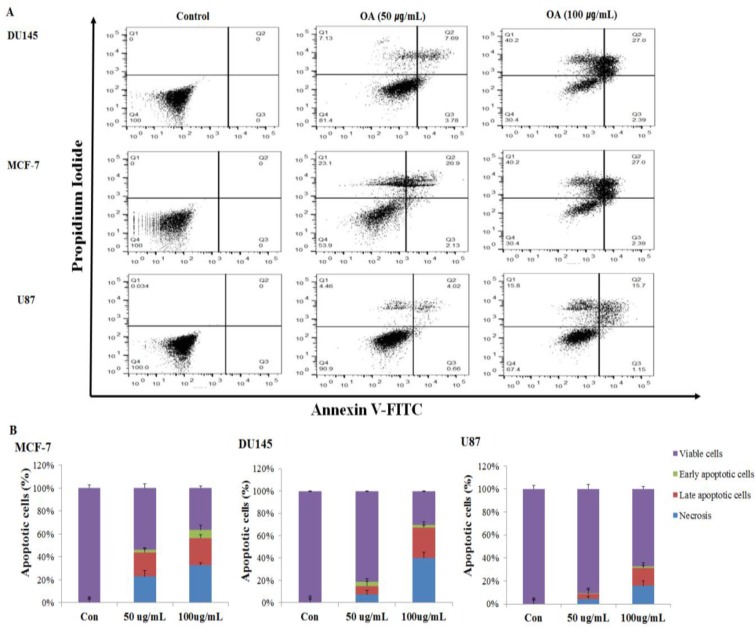
Dose dependent apoptotic cell death induced by oleanolic acid (OA) in DU145, MCF-7, and U87 cells (**A**) Annexin V-FITC/PI staining for the detection of apoptotic cells. After treatment with OA (0, 50, or 100 μg/mL), cells were stained with Annexin V-FITC/PI and subjected to flow cytometry. (**B**) Quantitation of the FACS data shown in (A). Results are expressed as mean ± SD.

To determine whether the anti-cancer effects of OA were associated with apoptosis, we treated DU145, MCF-7, and U87 cells with OA at 0, 50, and 100 µg/mL concentrations for 24 h. Apoptotic cells were quantified by Annexin V-FITC staining (Figure 1A). OA increased the number of late-apoptotic cells depending on the dosage. As shown in Figure 1B, treatment with 50 and 100 µg/mL OA resulted in 7.69% and 27.0% apoptotic cells in DU145, 20.9% and 27.0% apoptotic cells in MCF-7, and 4.02% and 15.7% apoptotic cells in U87 respectively, as compared to 0% (early-apoptotic and late-apoptotic cells) in the control. Our results showed that the OA treatment decreased cell proliferation and increased apoptosis in these cancer cell lines.

### Effect of OA treatment on the expression of apoptosis-related proteins

**Figure 2:**
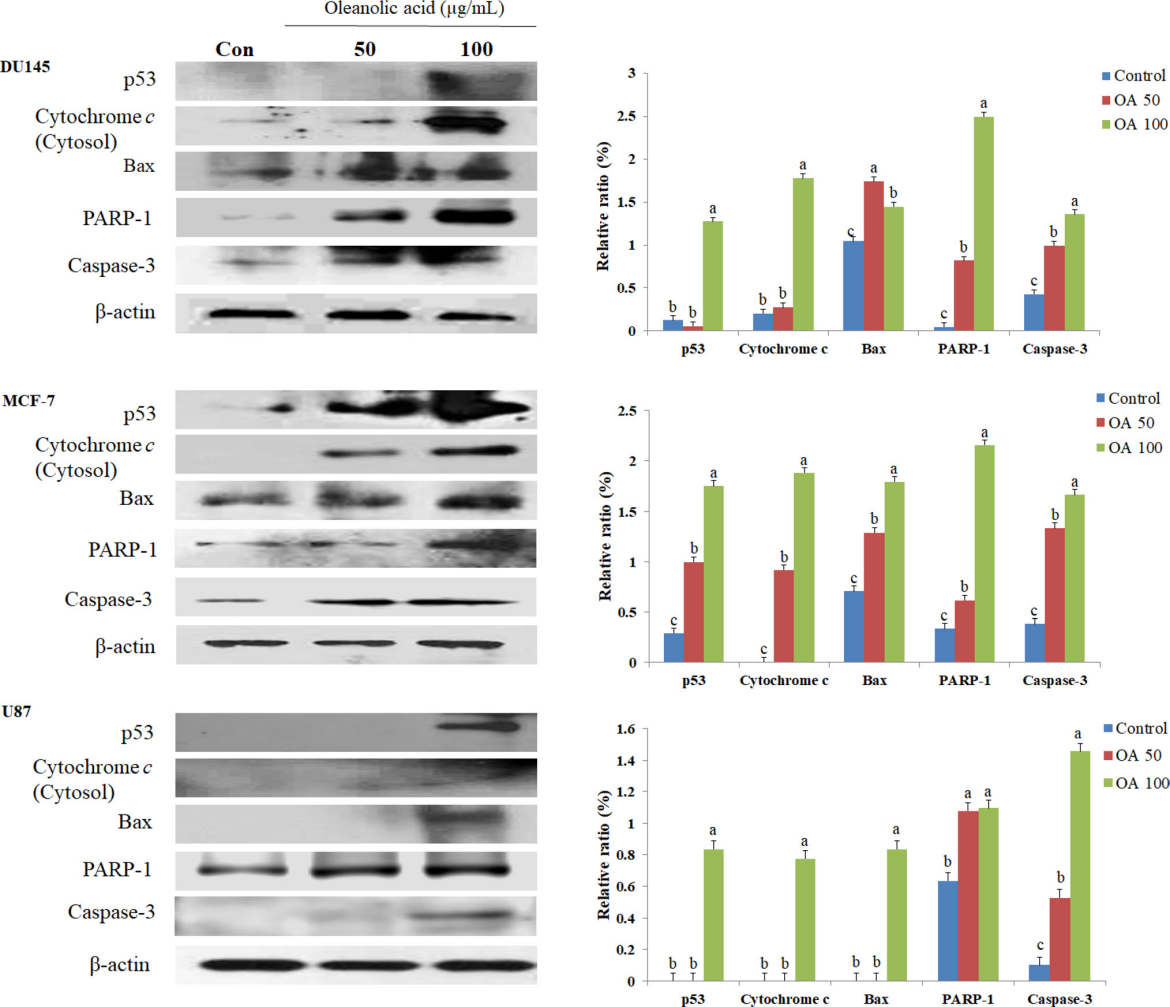
Effect of oleanolic acid (OA) on apoptosis in DU145, MCF-7, and U87 cells The cells were treated with 50 and 100 μg/mL OA for 24 h. After treatment, the production of p53, cytochrome c, Bax, PARP-1 and caspase-3 was determined by western blot. Equal loading was confirmed by β-actin quantification and the relative density of the proteins was measured and normalized to β-actin (arbitrarily set at 1). Results are expressed as mean ± SD. Significant differences (*p* < 0.05) are represented using different letters.

To characterize the molecular mechanism of OA-induced apoptosis in DU145, MCF-7, and U87 cells, we measured the expression of apoptosis-related proteins (p53, cytochrome c, Bax, caspase-3, and PARP-1) by western blot (Figure 2). OA treatment significantly increased the expression of apoptosis-related proteins in DU145, MCF-7, and U87 cells. The increased expression of p53 (10.11-fold) and cytochrome c (74.70-fold) in DU145 cells treated with 100 µg/mL OA was significantly higher than that of the control group. Moreover, the expression of Bax, PARP-1 and caspase-3 increased 1.51-, 51.59- and 3.16-fold respectively, in DU145 cells treated with 100 µg/mL OA as compared to that for the control group. Similarly, MCF-7 cells treated with 100 µg/mL OA showed increased expression of p53 (6.01-fold), cytochrome c(18.82-fold), Bax (2.53-fold), PARP-1 (6.39-fold) and caspase-3 (4.33-fold) compared with that observed for the control group. U87 cells treated with 100 µg/mL OA showed marked upregulation of p53, cytochrome c, Bax, PARP-1 and caspase-3. These results confirmed that apoptosis was induced in cancer cell lines treated with OA.

### Effect of OA on cell cycle progression

**Figure 3:**
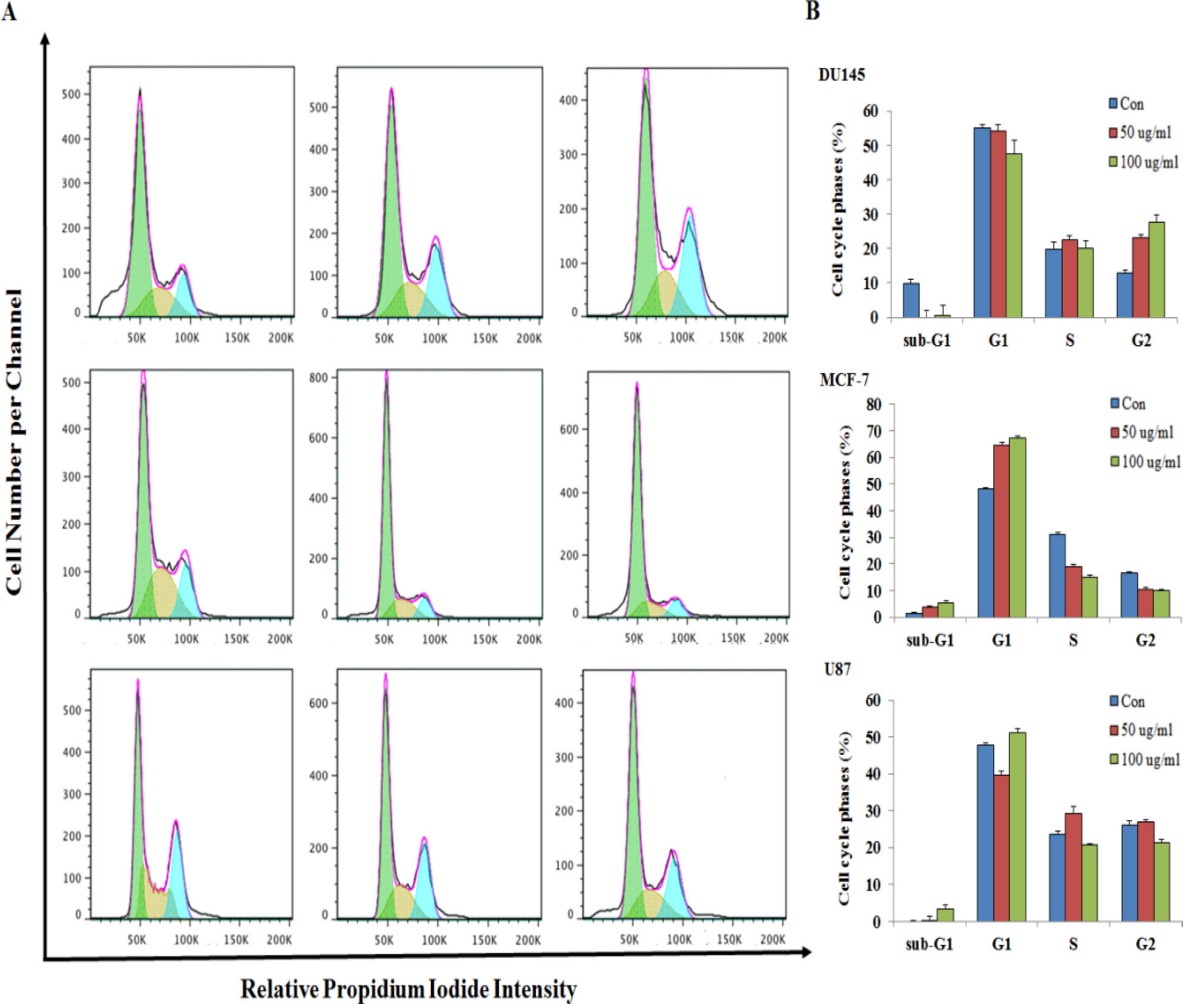
Effect of oleanolic acid (OA) treatment on cell cycle progression in DU145, MCF-7, and U87 cells (**A**) Flow cytometric analysis of cell cycle distribution in cells treated with 0, 50, and 100 μg/mL OA. (**B**) Histograms showing the percentages of cells in the G1, S, and G2 phases of the cell cycle. Results are expressed as mean ± SD.

To investigate the effects of OA on cell cycle progression, we treated DU145, MCF-7, and U87 cells with OA at 0, 50, and 100 μg/mL concentrations and analyzed the different stages of the cell cycle using flow cytometry Figure 3A. As shown in Figure 3B, OA-treated DU145 cells showed enhancement in the percentage of cells in G2 compared to the control cells (*P* < 0.05). Treatment with 50 and 100 µg/mL OA resulted in 23.15% and 27.62% of cells arrested in the G2 phase, respectively. Conversely, MCF-7 cells treated with the same growth-suppressive concentrations of OA reported a decrease in the percentage population (64.62% and 67.21%, respectively), and a decrease in the S and G2 populations compared to those in the control. MCF-7 and U87 cells were arrested the G1 phase at 100 μg/mL concentration. These observations suggested that OA impacted cell cycle progression based on cell types.

### Effect of OA on the expression of cell cycle regulators

**Figure 4:**
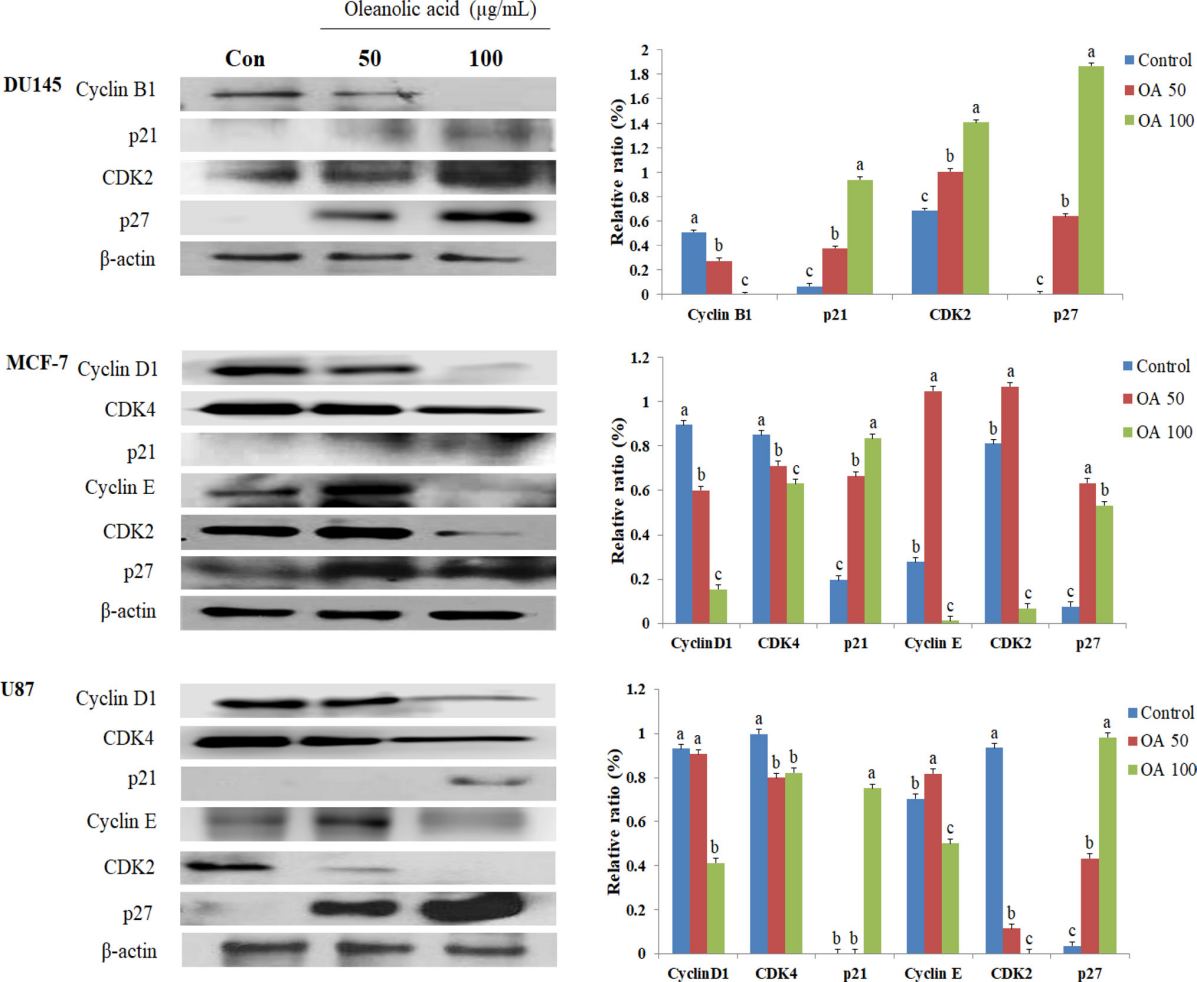
Effect of oleanolic acid (OA) treatment on the levels of cell cycle regulatory proteins in DU145, MCF-7, and U87 cells The cells were treated with 50 and 100 μg/mL OA for 24 h. After treatment, the expression levels of cyclin B1, cyclin D1, p21, cyclin E, CDK2, CDK4 and p27 were determined by western blot analysis. Equal loading was confirmed by β-actin quantification. For each protein, the relative density was measured and normalized to the β-actin bands (arbitrarily set at 1). The results are expressed as mean ± SD. Significant differences (*p* < 0.05) are represented using different letters.

As shown by western blot analysis in Figure 4, OA caused the arrest of cells in the G2 phase in DU145 cells and the G1 phase in MCF-7 and U87 cells. We then assessed its effects on the G1 and G2 cell cycle regulators cyclin B1, cyclin D1, cyclin E, CDK2, CDK4, p21, and p27. Treatment of DU145 cells with 100 µg/mL OA produced a marked decrease in the production of cyclin B1 when compared with that of the control. The same treatment produced increases in the levels of p21 (13.64-fold), p27 (377.19-fold), and CDK2 (2.05-fold). In MCF-7 cells, treatment with 100 µg/mL OA decreased the production of cyclin D1, cyclin E, CDK2, and CDK4 by 5.87-, 23.43-, 12.02-, and 1.34-fold, respectively, when compared with that reported for the control group, while simultaneously increasing the production of p21 (4.26-fold) and p27 (6.95-fold). Treatment of U87 cells with 100 µg/mL OA significantly increased the production of p21, and p27, while decreasing the levels of cyclin D1, cyclin E, CDK2, and CDK4.

### Effect of OA on ERK, JNK, and AKT activation in cancer cells

**Figure 5:**
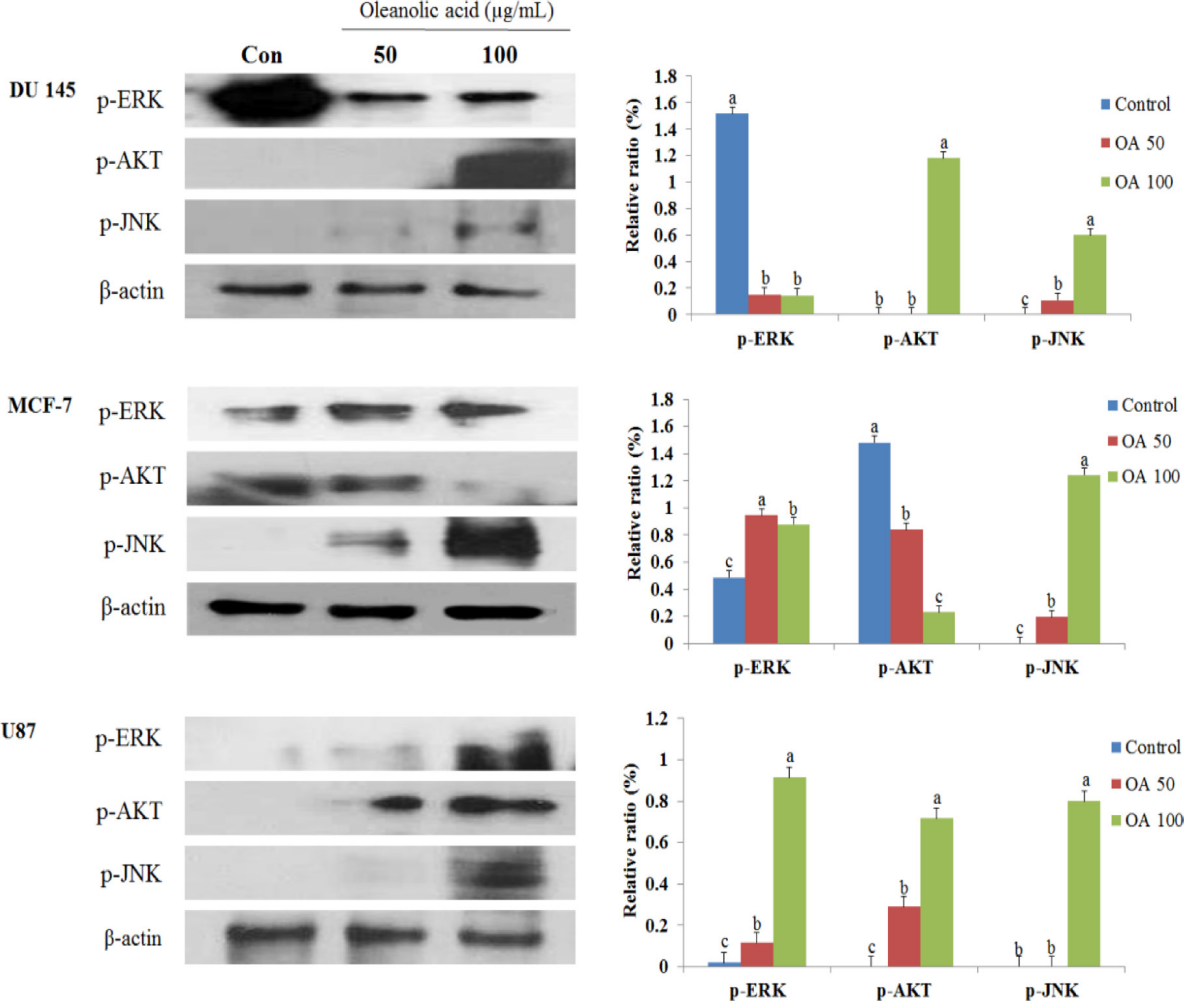
Effect of oleanolic acid (OA) on MAPK kinase signaling in DU145, MCF-7, and U87 cells The cells were treated with 50 and 100 μg/mL OA for 24 h. After treatment, the expression of p-ERK, p-AKT, and p-JNK was determined using western blot analysis. Equal loading of proteins was confirmed using β-actin quantification. Results are expressed as mean ± SD. Significant differences (*p* < 0.05) are represented using different letters.

To determine whether OA affects MAPK activation, we measured ERK, JNK, and AKT activation in response to OA in DU145, MCF-7, and U87 cells. As shown in Figure 5, the phosphorylation levels of JNK and AKT increased after OA treatment in DU145 cells, while the phosphorylation levels of ERK decreased. In MCF-7 cells, the levels of p-AKT significantly decreased, while those of p-ERK and p-JNK increased. Finally, an increase in p-ERK, p-AKT, and p-JNK expression levels was observed following the 100 µg/mL OA treatment in U87 cells. Our results demonstrated that the expression of proteins involved in MAPK kinase signaling was differently regulated in various cell lines.

### The inhibition of the MAPK signaling pathway is involved in the OA-induced anticancer effect

**Figure 6:**
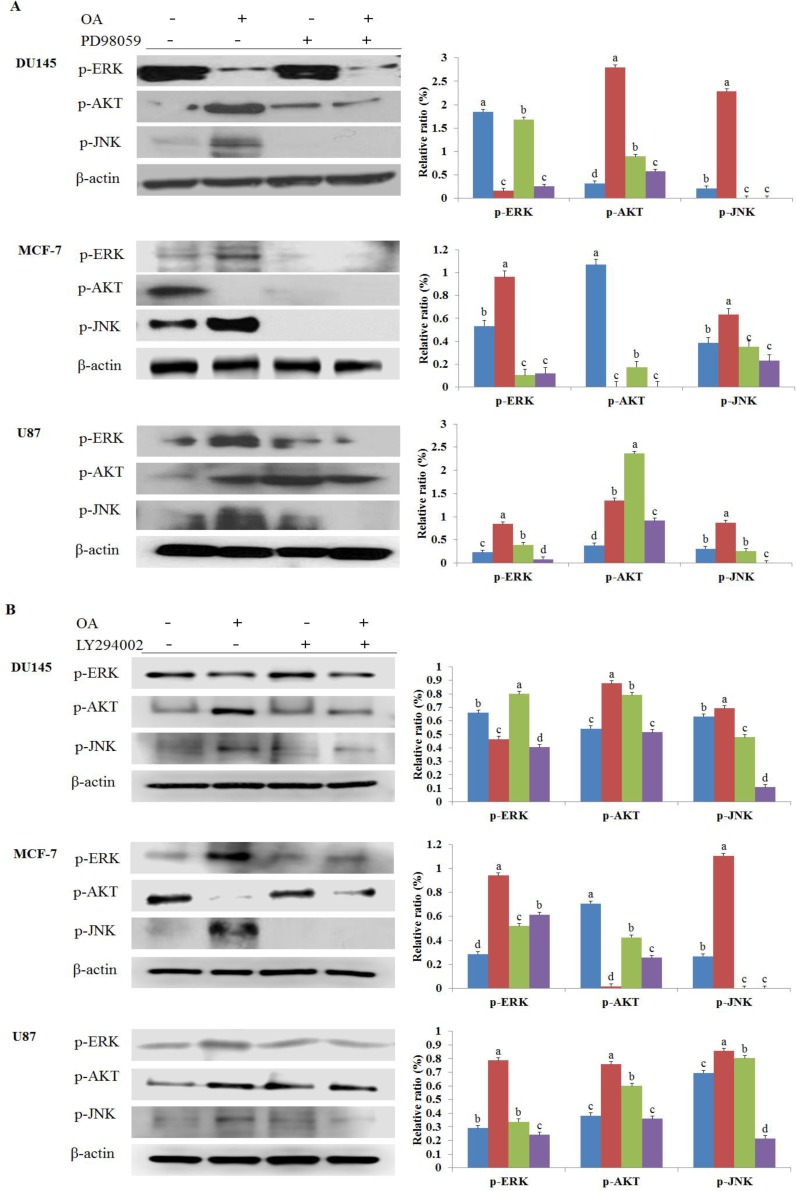
(**A**) The lone and/or combined effects of oleanolic acid (OA) and PD98059 on the expression of proteins involved in MAPK kinase signaling in DU145, MCF-7, and U87 cells. The cells were treated with OA (100 μg/mL) and PD98059 (50 μM), alone or in combination, for 24 h and the expression of p-ERK, p-AKT, and p-JNK was determined by western blot. (**B**) The lone and/or combined effects of oleanolic acid (OA) or LY294002 on the expression of proteins involved in MAPK kinase signaling in DU145, MCF-7, and U87 cells. The cells were treated with OA (100 μg/mL) and LY294002 (10 μM), alone or in combination, for 24 h and the expression of p-ERK, p-AKT, and p-JNK was determined by western blot. Equal loading of proteins was confirmed using β-actin quantification. Results are expressed as mean ± SD. Significant differences (*p* < 0.05) are represented using different letters.

To further test whether the inhibition of the ERK signaling pathway was involved in OA-induced anticancer activities in DU145, MCF-7, and U87 cell lines, the cells were treated with PD98059 (a selective inhibitor of MAPK) and LY294002 (AKT inhibitor) that disrupted the downstream expression of p-ERK, p-AKT, and p-JNK. As shown in Figure 6A, treatment with PD98059 increased the activation of p-ERK in DU145 cells when compared to that observed with OA only treatment. Moreover, treatment with PD98059 alone showed that the p-AKT and p-JNK levels decreased in DU145 cells. Combination treatment with PD98059 and OA further decreased the p-ERK and p-JNK levels in DU145 cells when compared to those observed after treatment with PD98059 alone. In DU145 cells treated with a combination of PD98059 and OA p-JNK levels did not change when compared to its levels following treatment with PD98059 alone. We found that treatment with PD98059 in MCF-7 cells reduced the expression of p-ERK and p-JNK compared to that detected with OA alone. However, no changes were observed in p-ERK, p-AKT, and p-JNK levels in MCF-7 cells treated with a combination of PD98059 and OA when compared to their levels in cells treated with PD98059 alone. In U87 cells treated with PD98059, p-ERK and p-JNK expression decreased when compared to cells treated with PD98059 alone. However, the expression of p-AKT increased in U87 cells treated with PD98059. The expression of p-ERK, p-AKT, and p-JNK in U87 cells treated with a combination of PD98059 and OA was lower than that observed in cells treated with PD98059 alone.

As shown in Figure 6B, treatment with LY294002 alone reduced the p-AKT and p-JNK levels in DU145 cells when compared with OA alone. However, treatment with LY294002 only increased the p-ERK levels in DU145 cells. Combination treatment with LY294002 and OA decreased the p-ERK, p-JNK and p-AKT levels in DU145 cells when compared to that observed with LY294002 alone. In MCF-7 cells treated with LY294002 only, p-ERK and p-JNK decreased compared to that detected with OA alone. Combination treatment with LY294002 and OA further increased the p-ERK levels in MCF-7 cells when compared to those observed after treatment with LY294002 alone. However, we found that treatment with LY294002 and OA in MCF-7 cells decreased the expression of p-AKT compared to that detected with OA alone. Also, MCF-7 cells treated with a combination of LY294002 and OA did not change in p-JNK levels when compared with LY294002 alone. The level of p-ERK, p-AKT, and p-JNK in U87 cells treated with LY294002 alone was lower than that observed in cells treated with OA alone. In U87 cells treated with a combination of LY294002 and OA, expression levels of p-ERK, p-AKT and p-JNK reduced when compared to their expression levels in cells treated with LY294002.

### PD98059 and LY294002 enhanced the OA-induced apoptosis in U87 cells

**Figure 7:**
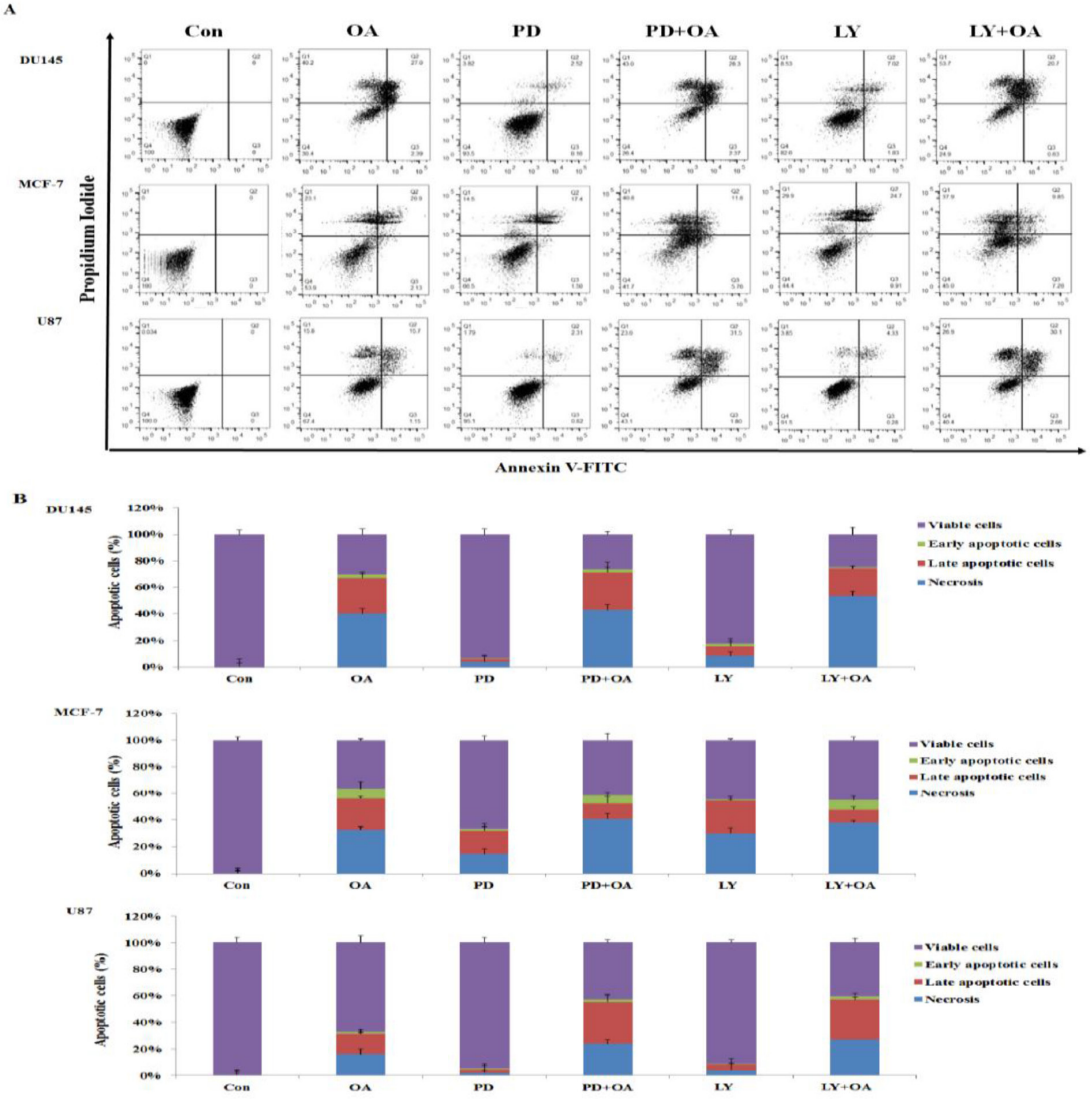
Apoptotic cell death in DU145, MCF-7, and U87 cells treated with oleanolic acid (OA) and/or PD98059 and/or LY294002, alone or in combination (**A**) Annexin V-FITC/PI staining for the detection of apoptotic cells. After treatment with OA (100 μg/mL) and/or PD98059 (50 μM) and/or LY294002 (10 μM), alone or in combination, cells were stained with Annexin V-FITC/PI and subjected to flow cytometry. (**B**) Quantitation of the FACS data shown in (A). Results are expressed as mean ± SD.

To determine whether the anti-cancer effects of OA were associated with apoptosis, we quantified apoptosis in DU145, MCF-7, and U87 cells with Annexin V-FITC staining using cells either individually treated with OA, PD98059, or LY294002, or in a combination of OA and PD98059 or OA and LY294002. As shown in Figure 7, treatment with PD98059 and LY294002 in DU145 cells did not affect apoptosis, when compared to the control. The number of late-apoptotic cells was also observed to decrease in PD98059 and OA combination-treated and LY294002 and OA combination-treated DU145 cells when compared to cells treated with OA alone. Moreover, the number of late apoptotic cells in MCF-7 cells treated with PD98059 alone was higher than that in control. In PD98059 and OA combination-treated and LY294002 and OA combination-treated MCF-7 cells, the number of apoptotic cells was lower than that in cells treated with PD98059 and LY294002 alone. However, in U87 cells treated with a combination of PD98059 and OA and LY294002 and OA, the number of late-apoptotic cells increased compared with that in cells treated with OA. Our results showed that pretreatment with the PD98059 MAPK inhibitor and LY294002 AKT inhibitor significantly increased the OA-induced apoptosis in U87 cells, but significantly decreased the OA-induced apoptosis in MCF-7cells. Quantitative analyses demonstrated that the synergistic effect of the PD98059 and LY294002 inhibitors manifested differently in various cell types.

### The OA-induced G1, S, and G2 phase arrest was due to the inhibition of the MAPK signaling pathway

**Figure 8:**
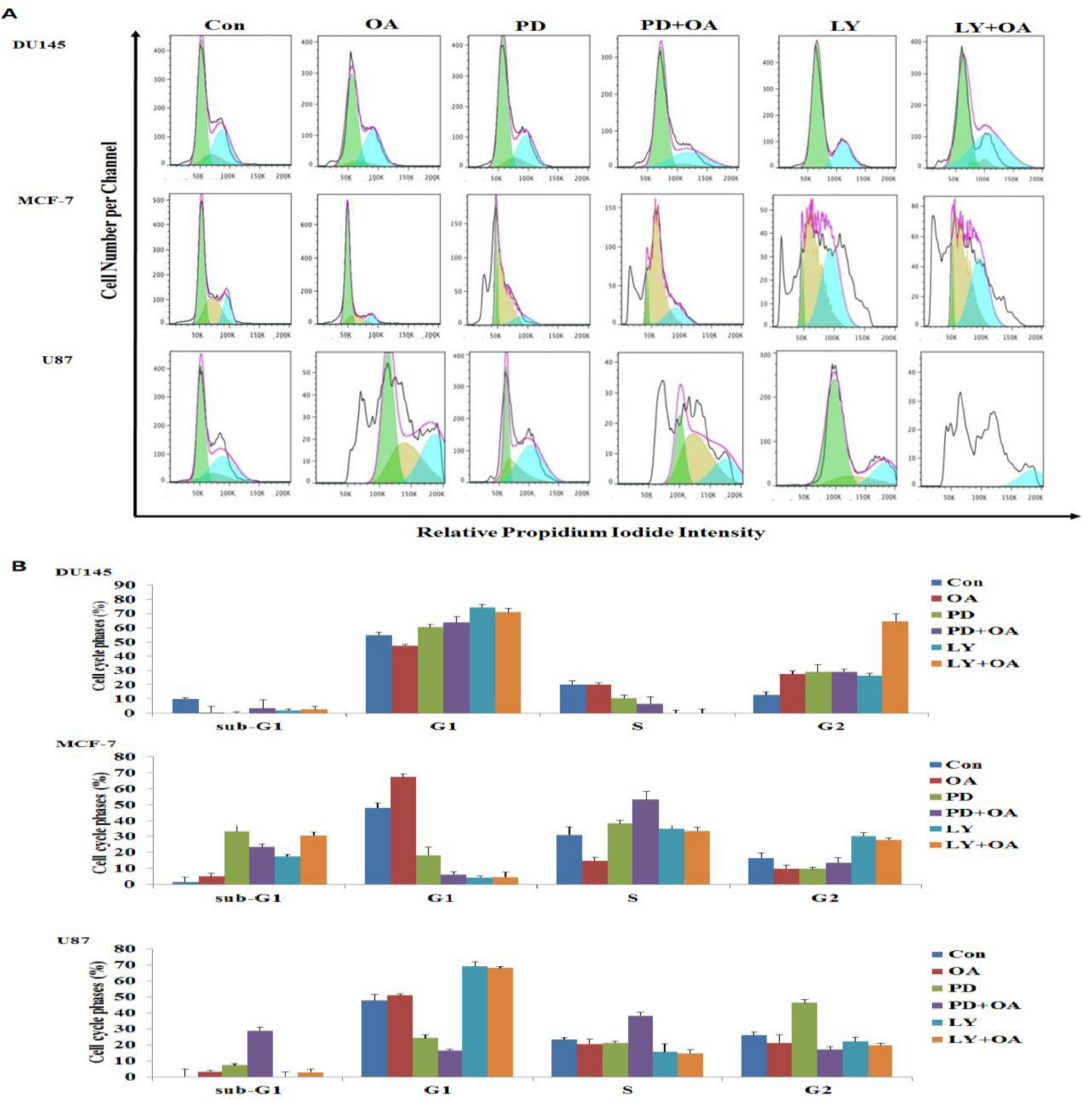
Effect of oleanolic acid (OA) and/or PD98059 and/or LY294002, alone or in combination, on the cell cycle distribution of DU145, MCF-7, and U87 cells The cells were treated with OA (100 μg/mL) and/or PD98059 (50 μM), and/or LY294002 (10 μM), alone or in combination, for 24 h, and the cell cycle distribution was estimated using flow cytometry. (**A**) Flow cytometry analysis of the cell cycle distribution in cells treated with OA (100 μg/mL) and/or PD98059 (50 μM) and LY294002 (10 μM), alone or in combination (**B**) Histograms showing the percentage of cells in the G1, S, and G2 phases of the cell cycle. Results are expressed as mean ± SD.

To investigate whether the inhibition of the ERK signaling pathway altered the distribution of cells in distinct phases of the cell cycle, we treated DU145, MCF-7, and U87 cells either individually with OA, PD98059, or LY294002, or in a combination of PD98059 and OA or LY294002 and OA, and eventually analyzed the cells using flow cytometry. As shown in Figure 8, treatment with PD98059 in DU145 cells increased the percentage of cells in the G1 phase as compared to that in DU145 cells treated with OA alone. Moreover, the number of cells in the G1 phase increased in DU145 cells treated with a combination of PD98059 and OA when compared to cells treated with PD98059 alone. DU145 cells treated with a combination of LY294002 and OA significantly elevated the percentage of cells in the G2 population in OA-treated cells from approximately 20.1% up to 62.3%. A higher percentage of cells arrested in the S phase were found following treatment with PD98059 alone, or with a combination of PD98059 and OA in the MCF-7 cell line. The population of cells in sub-G1 phase also increased in the LY294002 and OA combination treatment in MCF-7 cell line when compared to the LY294002 treatment. We also noted that U87 cells treated with PD98059 alone showed a significant increase in the number of cells in the G2 phase of the cell cycle, while the PD98059 and OA combination treatment enhanced the percentage of cells in the S phase when compared against the cells treated with PD98059 alone. These results also demonstrated that LY294002 treated U87 cells did not affect the cell cycle.

### OA increased antitumor effect in DU145 xenograft tumor model

**Figure 9:**
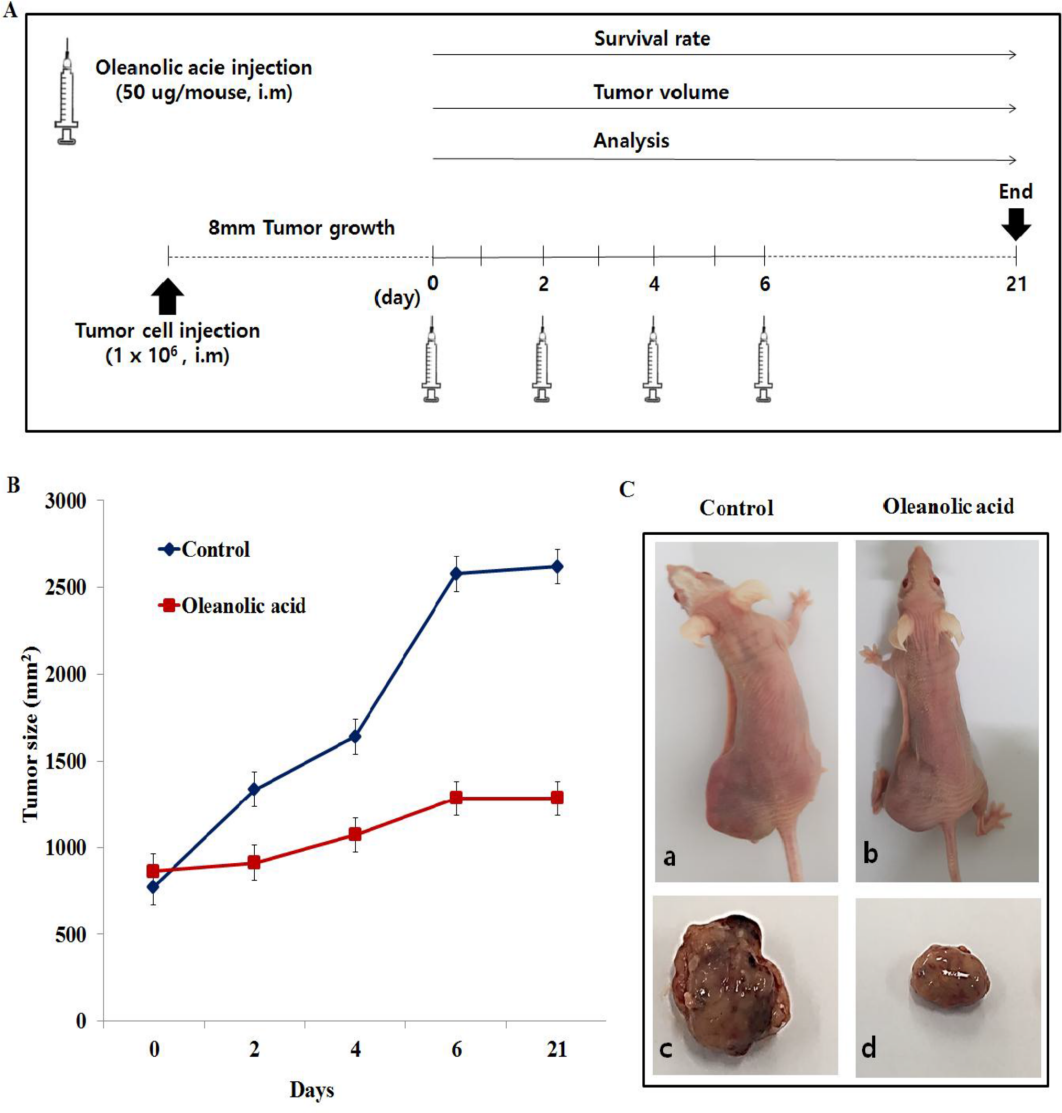
Oleanolic acid (OA) increased antitumor effect in DU145 xenograft model (**A**) Experimental design for OA treatment. (**B**) Tumor growth was measured twice per week and the tumor size was shown as mean size ± S.D. ^*^highlighted values with *P* < 0.05 when compared with the control group. (**C**) Tumor bearing mice (a: control, b: oleanolic acid) and the excised tumor tissues (c: control, d: oleanolic acid).

The above results showed that OA led to the enhancement of DU145 tumor cell expression *in vivo* and *in vitro*. DU145 cells (2 × 10^6^) were inoculated intramuscularly into the left things of mice and a treatment was initiated (a total of 4 injections of OA in 2 day intervals) when the tumor reached approximately 10 mm in mean diameter (Figure 9A). The antitumor effect was determined by the tumor growth delay and final tumor size (Figure 9B). Tumor growth of OA was significantly suppressed when compared with control groups (Figure 9B). The final average tumor size of OA treatment and control groups reported to be 1285 and 2618 mm^2^ respectively. Figure 9C showed that OA reduced about 29.38% average wet weight of tumors as compared to the control group.

### OA regulates apoptosis and cell cycle proteins and inhibits ERK/AKT/JNK activation in tumor tissues

**Figure 10:**
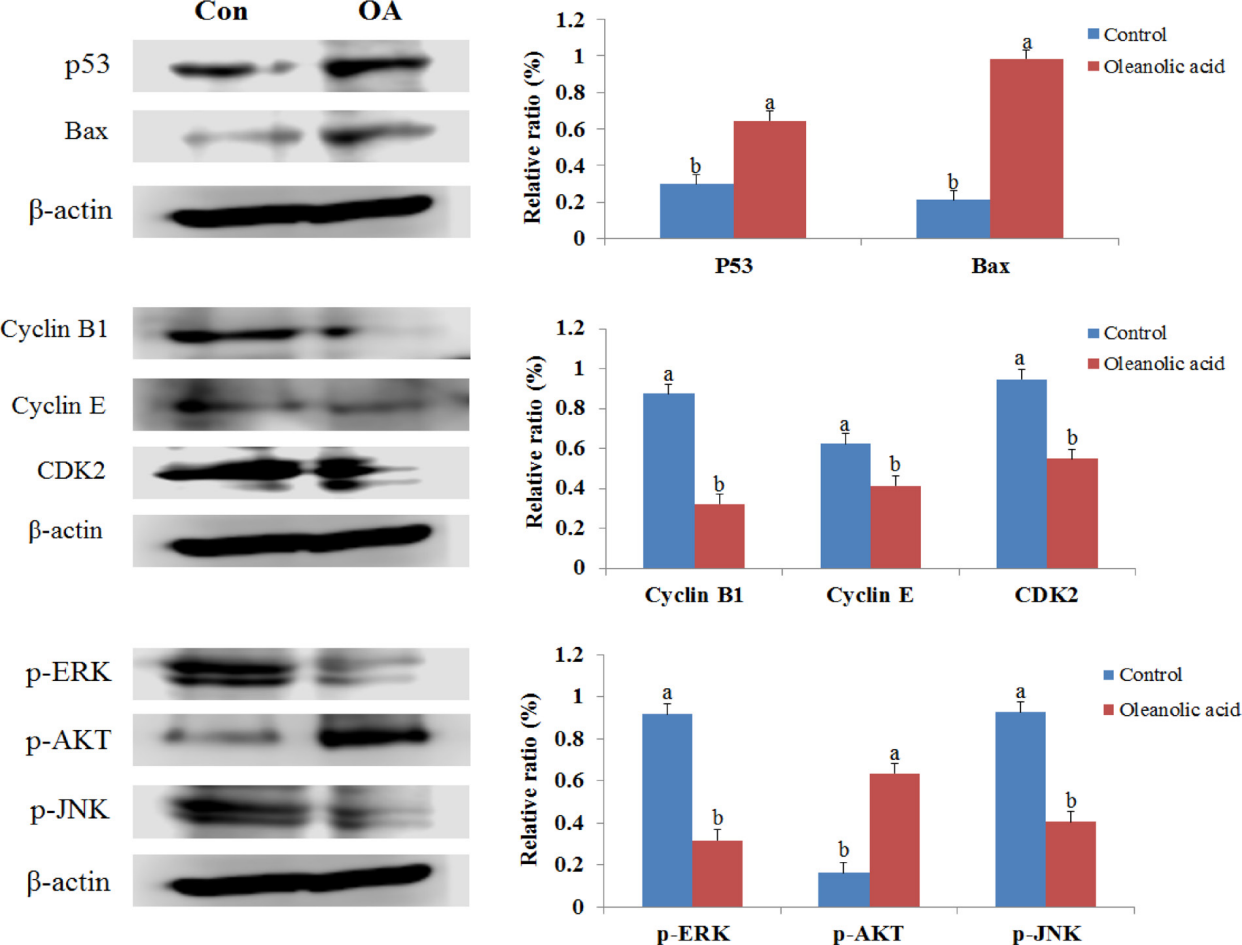
Effect of oleanolic acid (OA) on apoptosis, cell cycle-related proteins and ERK/AKT/JNK pathways in DU145 xenograft tumor tissues

Finally, we measured the effect of OA on the expression of apoptosis (p53 and Bax) and cell cycle proteins (cyclin B1, cyclin D, and CDK2) in tumor tissue by western blotting analysis (Figure 10). The expression of p53 and Bax increased 2.17- and 4.63-fold in OA treated groups when compared against the control group. OA also inhibited the expression of G1 and G2 cell cycle regulators such as cyclin B1, cyclin D, and CDK2 in xenografted tumors as compared to the untreated control group. These results suggested that OA possibly regulated tumor growth by causing cell cycle arrest and inducing apoptosis.

Previously, Figure 5 showed that the phosphorylation levels of JNK and AKT increased after DU145 cells were treated with OA. However, when DU145 cells were treated with 100 µg/mL OA, the expression of ERK considerably decreased when compared to the control. Therefore, here we looked to measure the expression of phosphor-ERK/AKT/JNK in tumor tissues (Figure 10). OA inhibited the expression of ERK/JNK in tumor tissues isolated from DU145 xenografts when compared against the untreated control group. However, the treatment with OA in tumor tissues increased the expression of AKT when compared to the control group. Overall these results suggested that the OA inhibited ERK/AKT/JNK pathway in DU145 xenografted tumors and that the inhibition of this pathway could induce cell cycle arrest, suppresses tumor cell proliferation, and prostate cancer growth.

## DISCUSSION

Our study investigated the effects of oleanolic acid (OA) on the viability, apoptotic activity, and cell cycle arrest of DU145 prostate cancer cells, MCF-7 breast cancer cells, and U87 human glioblastoma cells. We demonstrated that the treatment with OA inhibited cell proliferation in all of these cell lines. The IC50 values for the cytotoxic effects of OA were reported as 112.57, 132.29, and 163.60 µg/mL in the DU145, MCF-7, and U87 cell lines, respectively. Our results suggest that OA treatments affect cell cycle progression and apoptosis depending on the cell type.

Apoptosis in cancer cells is regulated through endonucleases and the sequential activation of a cascade of caspase enzymes. An increase in the activity of caspases is commonly observed in cells undergoing apoptosis [19, 20]. These enzymes, particularly caspase-3, bring about a series of characteristic changes associated with apoptosis. These changes include cell shrinkage, chromatin condensation, fragmentation, and plasma membrane blebbing [16]. Bax controls cell death through mitochondrial disruption, which leads to the release of cytochrome c into the cytosol [18]. The tumor suppressor gene p53 can be activated under a variety of conditions including hypoxia, heat shock, and DNA damage [16]. p53 regulates the transcription of genes associated with cell cycle arrest (e.g., Gadd45 and p21) and apoptosis (e.g., DR5, Bax, caspase-3, Apaf-1, Fas, p53-inducible gene, and Noxa) [16]. As depicted in Figure 1, apoptotic cells were detected in DU145 (27.0%), MCF-7 (27.0%), and U87 (15.7%) cell lines after treatment with OA. Moreover, OA treatment decreased cell viability and increased apoptosis in the prostate cancer cell line. The expression levels of the apoptosis-associated p53, cytochrome c, Bax, caspase-3, and PARP-1 proteins significantly increased in cancer cells treated with OA. These findings were consistent with a previous report of OA-induced apoptosis in human osteosarcoma cell lines (HOS, U2-OS and MG-63), human bladder cancer (T24 cells) and human gastric cancer (MKN28) [17, 31–32].

Induction of apoptosis and inhibition of cell proliferation are correlated with the activation of a variety of intracellular signaling pathways leading to cell cycle arrest in the G1, S, or G2/M phase of the cell cycle [33]. Key regulators of G1 progression in mammalian cells include three D-type cyclins (D1, D2, and D3), which assemble into holoenzymes with either CDK 4 or CDK 6 and cyclin E, which combines later in G1 with CDK 2. Overexpression of D- or E-type cyclins can contract G1, decrease cell size, and reduce the dependency of the cell on mitogens [34]. During the transition of the G1 to S phase, the association of CDK4 with D-type cyclins is critical for G1 phase progression, while p21 could prevent the cells from entering S phase [35, 36]. Cyclin B1 plays a key role in the cell cycle transition from the G2 to M phase [15]. Decreased expression of cyclin B1 may disrupt cell growth and promote malignant transcription [16]. Studies have demonstrated that cell cycle arrest at the G2/M phase by a DNA damaging agent is tightly associated with the induction of p21 [37–39]. p27 is another CDK member that can bind and inhibit a broader range of CDKs [37–38]. In the present study, we found that p27 and p27 induction by OA in DU145, MCF-7, and U87 cells is responsible for the downregulation of cylins and CDK expression.

Cell cycle regulation and its modulation by various cancer cells have been increasingly recognized and studied in recent years [37]. A large number of phytochemicals have been shown to inhibit cell cycle progression of various cancer cells [40]. The transition from the G0 to G1 phase of the cell cycle is regulated in part by a mitosis-promoting factor, which consists of cyclin D1 and CDK4 in gallbladder cancer cells [21]. In hepatocellular carcinoma, OA can induce G2/M cell cycle arrest, and the G2/M progression of the cell cycle is driven by the maturation promoting factor, a complex of cyclin B1/cdc2 [41]. Previous studies reported that OA treatments suppressed the percentage of cells in the S phase and enhanced the percentage of cells in the G0/G1 phase in HL-60 myeloid leukemia and sHCT15 colon carcinoma cell lines [21, 26**].** Additionally, gambogic acid from *Garcinia hanburyl* has also been reported to induce G2 arrest in BGC-823 human gastric carcinoma cells, similar to the effects we observed with OA [42]. Our cell cycle analysis demonstrated that OA treatments increased the percentage of G2 phase cells in DU145 cells. However, MCF-7 and U87 cells treated with growth-suppressive concentrations of OA showed increases in the number of cells in the G1 phase. p-JNK, p21, p27, and CDK2 protein levels increased and that of cyclin B1 decreased after treatment with OA in DU145 cells. Additionally, the expression levels of p-AKT, cyclin D1, cyclin E, CDK2, and CDK4 reduced while the levels of p21, p27, p-ERK and p-JNK increased in MCF-7 cells depending on dosage. Treatment of U87 cells with OA increased the expression of p21 and p27 proteins, while decreasing the protein levels of cyclin D1, cyclin E, CDK2, and CDK4. Our results demonstrate that the JNK signaling pathway modulated the mitochondrial pathway and caused G2 phase arrest in DU145 cells treated with OA. Furthermore, the results also suggest that the MAPK signaling pathway was required for the regulation of the p53-mediated G1 phase cell cycle arrest in MCF-7 cells. Interestingly, the effect of the OA treatment in our study differed from that in other reports. Our observations suggest that OA can differentially affect the cell cycle progression depending on the type of cell line.

AKT is a serine/threonine kinase. It is a key player in regulating cell signals that are important for cell death and survival [43]. Additionally, AKT is a master regulator involved in the transcriptional regulation of the anti-apoptotic protein Bcl-2, which plays a crucial role in prevention of cell death [44]. Activation of mitogen activated protein kinase (MAPK) signaling pathways is involved in the antiproliferative and proapoptotic effects of chemotherapeutics in many kinds of cancer cells [45, 46]. The effect of MAPK activation depends on the cell type as well as the stimulation and duration of activation. Stress-activated MAPKs are cancer-specific and play a sensitive role in drug therapy and outcome [47]. The MAPK signaling pathway comprises a family of protein kinases (JNK and ERK) that play important roles in cellular differentiation, proliferation, and survival [48]. Accumulating evidence indicates that activation of MAPK is associated with cell cycle arrest and induction of apoptosis [49]. Prolonged MAPK activation can induce p21 expression, leading to cell cycle arrest and cell senescence [50]. Several studies have suggested that oxidative stress can activate JNK and result in apoptosis [51]. JNK plays an essential role in activation of the intrinsic apoptotic pathway mediated by mitochondria in response to cellular stress, which facilitates translocation of the proapoptotic protein Bax to mitochondria when activated [52]. Moreover, ERK has also been identified to be involved in apoptosis and PARP signaling [53, 54]. Da-Wei Mu *et al.* reported that OA inhibited ERK1/2 and AKT in human bladder cancer (T24 cells) [31]. Additionally, a study using MKN28 gastric cancer cells reported that OA activated JNK, p38, and ERK, but inhibited AKT [32]. Others described the OA suppression mediated by the inhibition of the AKT, ERK, and p38 MAPK signaling pathways in SGC-7901, MGC-803, and BGC-823 gastric cancer cells [55]. In the present study, we demonstrated that the phosphorylation levels of JNK and AKT increased after OA treatment in DU145 cells. In MCF-7 cells, the levels of p-AKT significantly decreased, while those of p-ERK and p-JNK increased. In U87 cells, an increase in p-ERK, p-AKT, and p-JNK was observed following the 100 µg/mL OA treatment. Furthermore, the anti-cancer effects of OA *in vivo* were assessed. Our results showed that OA significantly inhibited the tumor growth of DU145 cells in BALB/c nude mice when compared with the control group, and that this effect was reversed by the overexpression of p-AKT, which was in accordance with our *in vitro* study. Collectively, the present data reveal that OA inhibits cell survival and proliferation of cancer cells, both *in vitro* and *in vivo*. OA could serve as a potential adjuvant for the treatment of cancer.

In conclusion, this study demonstrates that OA inhibits the growth of DU145, MCF-7, and U87 cancer cells and increases the expression of the apoptosis-promoting proteins p53, cytochrome c, Bax, caspase-3 and PARP-1. The cell cycle analysis showed that the OA treatment resulted in an accumulation of G2 phase cells, while concomitantly decreasing G1 phase cells in the DU145 cells. However, OA treatment of MCF-7 and U87 cells lines resulted in an accumulation of cells in the G1 phase, and a concomitant decrease in cells in the G2 phase. OA also led to increases in p-ERK and p21/WAF-1 protein expression and decreased cyclin B1 protein levels in DU145 cells. In MCF-7 cells, the expression of p21 and p53 increased, whereas those of cyclin D1, cyclin E, CDK2, CDK4, and p-AKT decreased in a dose-dependent manner. The anti-cancer effects of OA on cancer cells were orchestrated by the activation of apoptosis and limited cell cycle progression. These findings raise the possibility of using OA to effectively treat prostate and breast cancers, as well as cancers derived from other tissue types.

## MATERIALS AND METHODS

### Cell culture and reagents

Human prostate cancer (DU145), breast cancer (MCF-7), glioblastoma cancer (U87) and normal murine liver cell lines (BNL CL.2) were purchased from the Korean Cell Line Bank (Seoul, Korea). Human foreskin fibroblast (Hs 68) was obtained from American Type Culture Collection (ATCC, Rockville, MD). While U87, BNL CL.2 and Hs68 cells were grown in Dulbecco’s Modified Eagle Medium (DMEM), DU145 and MCF-7 cells were grown in RPMI-1640 culture medium (Welgene). DMEM and RPMI 1640 were supplemented with 10% fetal bovine serum (HyClone, Logan, UT), 100 U/mL penicillin, and 100 mg/mL streptomycin (GIBCO, Grand Island, NY, USA). Cancer cells were cultured as described in a previous study [56]. The cells were seeded into 96-well cell culture plates and allowed to grow for 24 h prior to treatment with oleanolic acid (Sigma, St. Louis, MO, USA).

### Cytotoxicity assays

The effects of OA on tumor cell proliferation were evaluated using the 3-(4, 5 dimethylthiazol-2-yl)-2, 5-diphenyltetrazolium bromide (MTT; Genetrone, Seoul, Korea) analysis.

### Measurement of apoptosis

Apoptotic cells were quantified using the Annexin V-FITC/PI double staining technique with an Annexin V-FITC apoptosis detection kit (BD Pharmingen, San Diego, CA, USA) according to the manufacturer’s instructions.

### Cell cycle analysis

Cells were seeded into 6-well plates (5 × 10^4^ cells/well) and treated with OA for 24 h, following which they were harvested, washed once with ice cold phosphate-buffered saline (PBS), and fixed with ice-cold 70% ethanol at 4° C. After removing the remaining PBS, cell pellets were suspended in 300 µL of propidium iodide (PI) solution (69 µM PI in 38 mM sodium citrate) and incubated at 37° C for 45 min in the dark. Cells were then analyzed using a flow cytometer (Becton Dickinson, CA, USA). The percentage of cell distribution was calculated using the CellQuest software. Specific protein inhibitors like PD98059 (PD) and LY294002 (LY) were purchased from Cell Signaling Technology (Beverly, MA, USA) and Calbiochem (San Diego, CA, USA) respectively.

### Animal experiments

All the experiments were conducted with the approval of the Institutional Animal Care and Use Committee at Konkuk University (IACUC approval number KU 17097), Seoul, Republic of Korea. Six- to 7-week-old male BALB/c mice were purchased from Orientbio Inc. (Seongnam, Gyounggi, Republic of Korea). All the mice were raised with free access to food and water under specific pathogen-free conditions in a room maintained under a 12 h light/dark cycle.

### Tumor growth experiments

Tumor volume was measured every 2 days with an electronic caliper and calculated as follows: *volume = π/6 × ab2*, where ‘a’ was the long axis and ‘b’ was the short axis of the two orthogonal diameters. The maximum permissible size of the tumors in mice was limited to 20 mm in diameter according to the IACUC guidelines at the Konkuk University. After carrying out the measurements, the experimental mice were sacrificed using carbon dioxide (CO_2_) before the tumors reached the maximum allowable size.

### Western blot analysis

Cells were lysed in ice-cold RIPA lysis buffer and 1 mM phenylmethylsulfonyl fluoride (protease inhibitor). The membranes were incubated with primary antibodies including p53 and p-AKT (Epitomics, Burlingame, CA, USA); cytochrome c, Bax, caspase-3, p-ERK, p-JNK, CDK2, p27, and b-actin (Cell Signaling Technology); p21 and cyclin B1 (Santa Cruz Biotechnology, Santa Cruz, CA, USA). The membranes were then incubated with a goat anti-rabbit IgG (H+L) HRP-conjugated secondary antibody (Zymax, San Francisco, CA, USA). The antigen-antibody complexes were visualized by enhanced chemiluminescence. Densitometric analysis of the signal was performed using a C-DiGit Blot Scanner (Li-COR Inc., Lincoln, NE, USA). Relative expression was quantified using Image J (NIH, Bethesda, Rockville, MD, USA) and compared to β-actin.

### Statistical analysis

The statistical analysis was performed using SPSS 18.0 (SPSS Inc., Chicago, IL, USA). Averages and standard deviations were calculated and differences between groups were assessed using the analysis of variance method and Duncan’s multiple range test. Differential values were considered significant if *p* < 0.05.
